# A computational framework for improving genetic variants identification from 5,061 sheep sequencing data

**DOI:** 10.1186/s40104-023-00923-3

**Published:** 2023-10-02

**Authors:** Shangqian Xie, Karissa Isaacs, Gabrielle Becker, Brenda M. Murdoch

**Affiliations:** 1 Department of Animal, Veterinary & Food Sciences, University of Idaho, Moscow, ID USA; 2 Superior Farms, California, USA

**Keywords:** Computational framework, Genetic variants, Multiple samples, Sheep

## Abstract

**Background:**

Pan-genomics is a recently emerging strategy that can be utilized to provide a more comprehensive characterization of genetic variation. Joint calling is routinely used to combine identified variants across multiple related samples. However, the improvement of variants identification using the mutual support information from multiple samples remains quite limited for population-scale genotyping.

**Results:**

In this study, we developed a computational framework for joint calling genetic variants from 5,061 sheep by incorporating the sequencing error and optimizing mutual support information from multiple samples' data. The variants were accurately identified from multiple samples by using four steps: (1) Probabilities of variants from two widely used algorithms, GATK and Freebayes, were calculated by Poisson model incorporating base sequencing error potential; (2) The variants with high mapping quality or consistently identified from at least two samples by GATK and Freebayes were used to construct the raw high-confidence identification (rHID) variants database; (3) The high confidence variants identified in single sample were ordered by probability value and controlled by false discovery rate (FDR) using rHID database; (4) To avoid the elimination of potentially true variants from rHID database, the variants that failed FDR were reexamined to rescued potential true variants and ensured high accurate identification variants. The results indicated that the percent of concordant SNPs and Indels from Freebayes and GATK after our new method were significantly improved 12%−32% compared with raw variants and advantageously found low frequency variants of individual sheep involved several traits including nipples number (*GPC5*), scrapie pathology (*PAPSS2*), seasonal reproduction and litter size (*GRM1*), coat color (*RAB27A*), and lentivirus susceptibility (*TMEM154*).

**Conclusion:**

The new method used the computational strategy to reduce the number of false positives, and simultaneously improve the identification of genetic variants. This strategy did not incur any extra cost by using any additional samples or sequencing data information and advantageously identified rare variants which can be important for practical applications of animal breeding.

**Supplementary Information:**

The online version contains supplementary material available at 10.1186/s40104-023-00923-3.

## Introduction

Genetic variation refers to differences in the genetic makeup of individuals in the same species. Single nucleotide polymorphisms (SNPs) and insertions/deletions (Indels) are two common types of genetic variants among individuals [1], which contribute to genetic diversity and critically influence phenotypic differences, including diseases susceptibility in human [2–4], trait enhancement and disease resistance in animal and plant breeding [5–7]. The genetic variants related to the phenotypes can be used to inform disease prediction [4], identification of causal mechanisms of disease [2], and the prioritization of biological targets in breeding programs in plants and animals [6, 7]. Improving productivity of animal or plant breeding will require a better understanding of the related genetic variants function in biological processes and how they interact with non-genetic components of production systems (e.g., nutrition and environment) [8, 9]. With the drastically decreasing cost of high throughput sequencing over the past decade, mass sequencing data have been used to support the understanding of genome to phenome (G2P). The accurate identification of genetic variants is a crucial point from mass sequencing data.

Several computational pipelines have been developed to analyze genetic variants, which mainly consist of the quality assessment, read alignment, variant calling, and functional annotation [10]. The performance of the specific part(s) or the whole analysis process were simultaneously improved with the development of suitable computational analysis tools. For the identification of SNPs and Indels, variant calling is core to the whole process or pipeline, and is conducted by variant callers based on sequence read alignment. At present, the mainstream variant callers include GATK [11–13], Freebayes [14], and SAMtools/BCFtools [15, 16]. GATK HaplotypeCaller is a tool to call SNPs and Indels via local de-novo assembly of haplotypes in an active region, which in some cases discards the existing mapping information and completely reassembles and realigns the reads in that region. This allows the HaplotypeCaller to be accurate within a region that contains different types of variants close to each other [12]. Freebayes is a bayesian genetic variant detector based on the sequences of reads aligned to a particular target, rather than the specific alignment [14]. It bypasses the problem of identical sequences that might align to multiple locations. BCFtools is a collection of several commands and generates the mpileup from the BAM alignment reads using SAMtools and then computes the variant calling by estimating mutation and sequencing error probabilities [15, 16].

Accurate identification of genetic variants plays a critical role in downstream analysis of G2P. Several factors contribute to the high accuracy of variant callings, including (1) Read quality (read sequencing error and read depth): a low sequencing error and a high read coverage of overlapping reads at the variant position support for high accuracy variants [17]; (2) Mapping quality: sequence reads aligned to a suitable and correct place in the genome sequence resulted in high mapping quality [18]. Recently, statistical models including Bayes [19] and Poisson [20, 21] were proposed to improve the mapping quality by incorporating base sequencing error into alignment; (3) Sample information: joint variant calling for multiple samples to allow mutual support of identified genotypes [8]; (4) Reference genome: a complete and high-quality reference genome can improve analysis of genetic variants [22].

Revolutionary next generation sequencing (NGS) technologies have remarkably decreased the cost of genome sequencing and lead to the brilliant achievements in genome sequencing projects such as the 1000 Genomes Project [23], the 1000 Bull Genomes Project [24] and the International Sheep Genomics Consortium 1000 sheep project (https://www.sheephapmap.org). Population genomic is recently emerging and facilitates a more comprehensive characterization of genetic variation in population-scale [25]. Population genomic approaches have now been used in many species to determine the effects of genetic variants [6, 7, 26, 27]. To date, joint calling is typically favored for population-scale genotyping as it generates a set of genetic variants [27]. For instance, variants calling in a population with GATK is performed by jointly calling from intermediate files (gVCF), which contain the candidate variant at each position of the genome. The identified variants of single sample are then combined across all samples to generate full variants for the population [11–13]. And Freebayes conducts the jointly calling for all samples present in the bam files using reads groups [14]. The jointly calling combines the variants from each sample without full utilization of multiple samples information from population.

Here we developed a computational framework for improving identification of genetic variants of 5,061 sheep from Flock54℠ program (https://www.flock54.com), which is a targeted genotyping panel that allows producers to test their flock's DNA for animal parentage and traits associated with disease, production and meat quality. The proposed framework incorporated the sequencing error and optimized mutual support information from multiple samples’ data in population scale. Firstly, the probabilities of variants identified from GATK and Freebayes were calculated by Poisson model incorporating base sequencing error potential for each single sample. Then the identified variants were ordered by probabilities and controlled by false discovery rate (FDR) using the construction of high-confidence identification dataset from multiple samples. The new method is illustrated through the high accuracy of variants called and the ability to detect variants even at low frequencies within the population of 5,061 sheep, which is predicted to have a profound impact on the identification of functional variants in biological processes or other studies.

## Materials and methods

### Workflow of variants identification

**Fig. 1:**
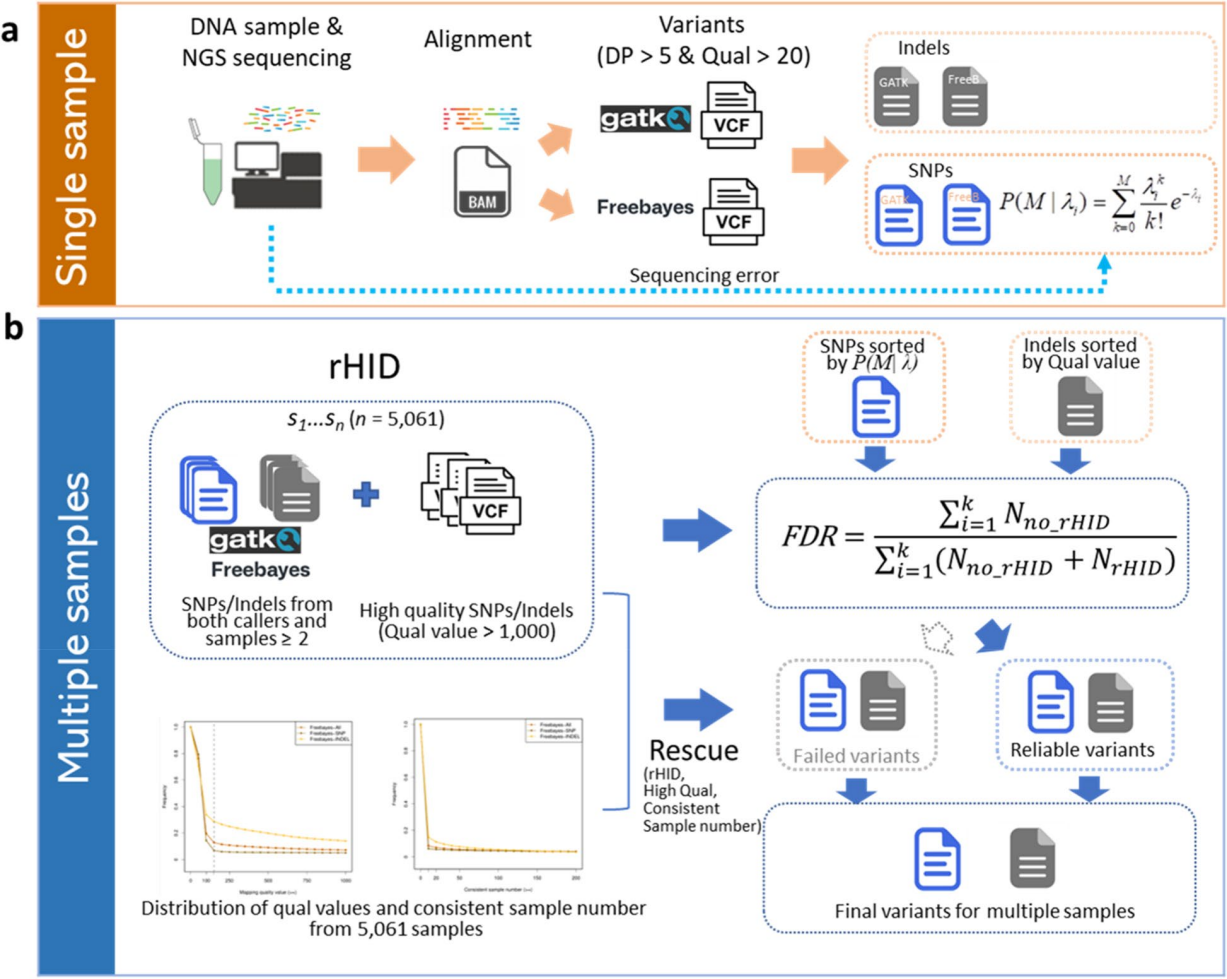
Workflow of the variant identification. **a** for a single sample; **b** for multiple samples

The workflow of the computational strategy for identifying variants from multiple samples consisted of four steps: (i) Probabilities of variants identified from GATK and Freebayes were calculated by Poisson model incorporating base sequencing error potential for each single sample (Fig. 1a); (ii) The variants with high mapping quality (> 1,000) or consistently identified from at least two samples by both callers (GATK and Freebayes) were used to construct the raw high-confidence identification variants database (rHID); (iii) The variants identified in single sample were ordered by probability value and controlled by FDR using rHID; (iv) To avoid the elimination of true variants from rHID, variants that failed after FDR were reexamined to identify any variants that might be rescued to ensure high accurate identification variants (Fig. 1b).

### Sequencing data analysis and variants calling

A total of 5,061 sheep tissue or blood samples were collected for the Flock54 program (https://www.flock54.com). Extracted DNA was sequenced by a targeted next-generation sequencing (NGS) panel using Thermo Fisher Ion Torrent platform as previous study’s description [28].

The sequencing quality of raw DNA short reads from 5,061 sheep were assessed and controlled by FastQC v0.11.6 (https://www.bioinformatics.babraham.ac.uk/projects/fastqc). Clean sequences were aligned to the latest reference genome ARS-UI_Ramb_v2.0 by using Bowtie2 v2.4.5 with default parameters [29]. The statistics of all mapped reads were calculated by flagstat of Samtools v1.15 [16], and the duplicate reads of alignment bam file were marked by MarkDuplicates of Picard v2.25.4 (http://broadinstitute.github.io/picard). And sample group information was added to bam files by AddOrReplaceReadGroups of GATK v4.1.7 [11]. Then the genetic variants were called by GATK v4.1.7 [11] and Freebayes v1.3.2 [14], respectively. The HaplotypeCaller, GenotypeGVCFs models of GATK were used to call variants for each sample with default parameters and generate vcf files with parameters -stand-call-conf 10. The Freebayes-parallel was used for fast parallel calling of SNPs and Indels for all 5,061 samples with default parameters. The multi-sample callers of GATK had the best accuracy particularly at 5× coverage depth if samples were called together with a large number of individuals such as those from 1000 Genomes Project in previous study [30]. All identified variants from GATK and Freebayes were filtered by vcftools with --minQ 20 --min-meanDP 5 [31], and the variants with high mapping quality (> 20) and reads coverage depth (> 5) were then extracted into separate SNP and Indel files by vcftools with parameters --remove-indels and --keep-only-indels, respectively.

For the construction of rHID, the filtered variants from each sample were merged by bcftools v1.9 [16], and the variant quality score of combined vcf files from GATK and Freebayes were recalibrated by VariantRecalibrator and ApplyVQSR [11], respectively. The recalibrated variants with quality higher than 1,000 or identified in both callers at least two samples were as the positive variants in rHID.

### Poisson model for SNP identification incorporating sequencing error

To distinguish the single position variant from the sequence error, the Poisson model of incorporating sequencing error was conducted with parameters *λ* and *k* (Fig. 1a). The Poisson cumulative distribution function that there is an actual mutation at a particular position is defined as follows:

$$P\left(M\right|{\lambda }_{i})={\sum }_{k=0}^{M}\frac{{\lambda }_{i}^{k}}{k!}{e}^{-{\lambda }_{i}}$$

Where *M* is the reads number that support a mutation for alternative allele at the *i*^th^ position, and ${\lambda }_{i}$ is the expect reads number with the platform sequencing error in the *i*^th^ position, ${\lambda }_{i}={N}_{i} \times {r}_{i}$, where *N*_*i*_ is the total number of reads covering the *i*^th^ position and *r*_*i*_ is the average sequencing error by calculating the phred score Q of *N* reads, *r*_*i*_ = ${10}^{-Q/10}/N$, as description in previous study [32]. For one single sample, the observed count of the alternative allele at *i*^th^ position supports the true variant if *P*(*M*|*λ*_*i*_) stays above a certain threshold. To avoid increasing type I error for multiple samples, all probabilities of identified variants were calculated in each single sample and the final threshold value was determined by the rHID information from multiple samples.

### Variant identification from multiple samples

False discovery rate (FDR) control is the most common method for assuring the overall quality of the set of identifications [33]. The false positive of variants in each sample can be controlled by the global positive variants from multiple samples, which provide extra and cross validation information for high confidence identification of variants. The FDR was defined by the expected value of the following formula:

$$FDR=E\left[\frac{F}{F+T}\right]$$

Where *F* and *T* are the expected number of false positive and true positive variants in each sample, which were based on the global positive variants from multiple samples.

The procedure of FDR control for the identification of high confidence variants from multiple samples is:

1. Construction of rHID from multiple samples (Fig. 1b). The rHID consists of three parts: (1) the variant with mapping quality > 1,000 from GATK, (2) the variant with mapping quality > 1,000 from Freebayes, and (3) identified in both callers and in at least two samples.
2. Marking positive or negative for the identified variants in an individual sample. The variants from an individual sample that cross validated in rHID were marked as positive. Conversely, the variants that were absent from the rHID were marked as negative for this individual sample.
3. Calculation of FDR values for all identified variants in an individual sample. Poisson probabilities of *n* SNPs were sorted by descending in an individual sample. FDR of the *i*^th^ SNP was calculated by *FDR*_*i*_*= F*_*i*_*/(F*_*i*_*+T*_*i*_*) =*$\sum _{1}^{i}\left(negative\ variants\ number\right)/\sum _{1}^{i}\left(total\ variants\ number\right)$. With a FDR > 0.01 threshold, variants from 1 to (*i-*1)^th^, those that were below the threshold of FDR, were regarded as Reliable variants (RVar), and the variants from *i*^th^ to *n*^th^, those that were above the threshold of FDR, were regarded as Failed variants (FVar). This was done to identify the few variants that may originally have been identified as negative but have a high enough quality value to pass the 1% FDR threshold and therefore should be retained. Furthermore, a few variants originally identified as positive but with a low quality value will not pass the 1% FDR and not retained. For Indels, mapping qualities of variants were sorted by descending, other parameters were similar to those used in SNPs.
4. Rescue variants. To avoid the elimination of true variants for an individual sample, the mapping quality (MQ) and consistent sample number (SN) of variants from all the samples were assessed and used to rescue the FVar of single sample with high *MQ* and *SN*. The 150 (Freebayes) and 300 (GATK) of *MQ* and 10 of *SN* were used as threshold values (Fig. S1). In the RVar group, the negative variant with mapping quality less than *MQ* and sample consistency less than *SN* is removed. In the FVar group, the positive variant with mapping quality more than *MQ* and sample consistency more than *SN* is rescued into final identified variants (FIV), and the unlisted variants of FIV that identified in raw variants were marked as final removed variants (FRV).

### Validation of variants identification from multiple samples

To illustrate and validate the reliability and rationality of the final identified variants from multiple samples, three metrics were used to validate the process of computational workflow: (1) Poisson probability: the variants from all sheep samples were divided into two groups (Pro = 1 and Pro < 1) according to the probabilities from Poisson model incorporating sequencing error, then the sequencing error and sequence depth were compared between these two groups. (2) Comparison of concordance variants in raw and FIV: concordance variants from GATK and Freebayes regarded as high confidence variants were compared between raw variants and FIV. (3) Comparison of variants in FDR control process: The mapping quality of variants from pre- and post-FDR were compared by eight groups: negative variants in pre-FDR (Neg), positive in pre-FDR (Pos), FIV, FRV, positive in both pre- and post-FDR (pos-FIV), negative in both pre- and post-FDR (neg-FRV), positive in pre-FDR but negative in post-FDR (pos-FRV), negative in pre-FDR but positive in post-FDR (neg-FIV).

### Properties of identified FIV from multiple samples

The FIV originally identified from freebayes and GATK were independently listed and marked as variant *type|caller* (SNP:INDEL|GK:FB) from the union variants of both two callers. The FIV then were divided into three groups based on the sample’s information: high, medium, and low frequency variants. The high frequency variants were those that were presented in more than 90% of all samples (4,555), and low frequency variants were uniquely identified in a single individual, all other variants were considered of medium frequency. Based on multiple samples, the common and specific variants can be accurately identified, especially low frequency variant identification in population scale. To further confirm and explain the functions of identified specific variants of multiple samples, the distribution and related genes of low frequency variants from single sheep were cross validated by IGV (Integrative Genomics Viewer) [34] and the knowledge of biological function described from previous studies. All variants were annotated by custom scripts based on the annotation GFF file of reference genome ARS-UI_Ramb_v2.0 [35]. The custom scripts and code can be found in github (https://github.com/shang-qian/Multi_Var).

## Results

### Overview of sequencing data

**Fig. 2:**
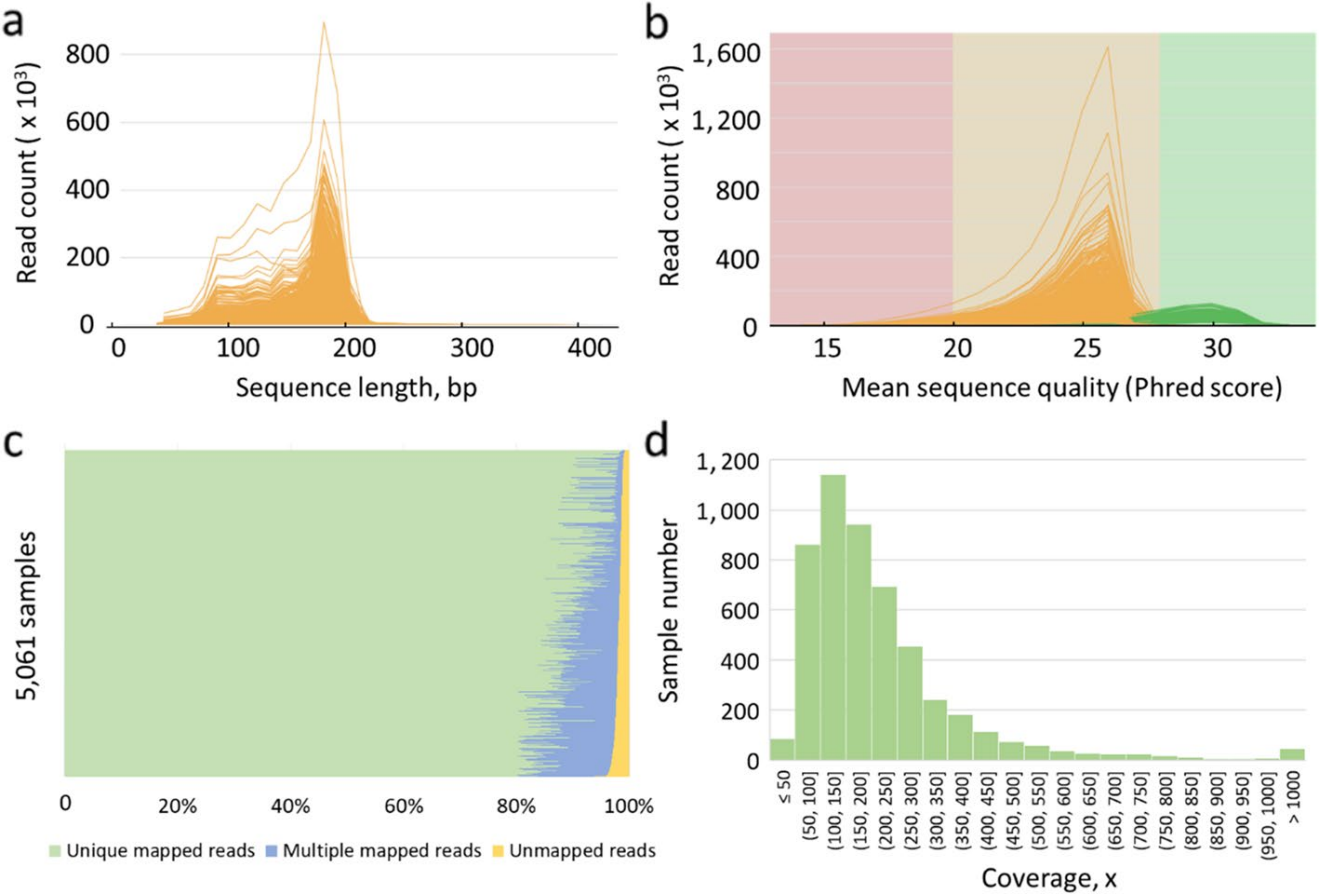
Statistical information of sequencing data from 5,061 samples. **a** Sequence length distribution; **b** Sequence quality distribution; **c** Unique and multiple mapped reads; **d** Average sequencing coverage for samples

A total of 5,061 sheep from Flock54 program were sequenced by targeted sequencing panel of short reads platform (Table S1). The length distribution of all sequences ranged from 50 to 400 bp, and most of them are distributed on the 100–200 bp (Fig. 2a) and the average sequence length of all samples is 152 ± 8 bp (Table S1). There were 207,732,536,962 bp total sequence length from 1,359,591,019 total reads in 5,061 samples, which covered a 171,567 ± 8,532 bp target sequencing region (> 5×) of the panel (Table S1). The min, max and average sequencing error in all samples were 0.00439, 0.01268 and 0.00842, respectively (Table S1). And the average sequencing quality of all samples were higher than phred score 20 (1% sequencing error rate), and some of them were even more than 30 (Fig. 2b), which met the requirement of quality control for sequencing reads. The high-quality reads were then aligned to reference ARS-UI_Ramb_v2.0. The average mapped reads rate was 98.11% ± 0.62%, most of which were uniquely mapped reads (the minimum and average unique mapped rate were 80.09% and 95.60%) (Fig. 2c and Table S1). The min, max and average coverage of mapped reads in targeted sequencing regions were 16, 3,473 and 216, respectively. And the majority of samples (more than 2,000) were mainly distributed between 100 and 200 (Fig. 2d).

### Probability from Poisson model incorporating sequencing error

**Fig. 3:**
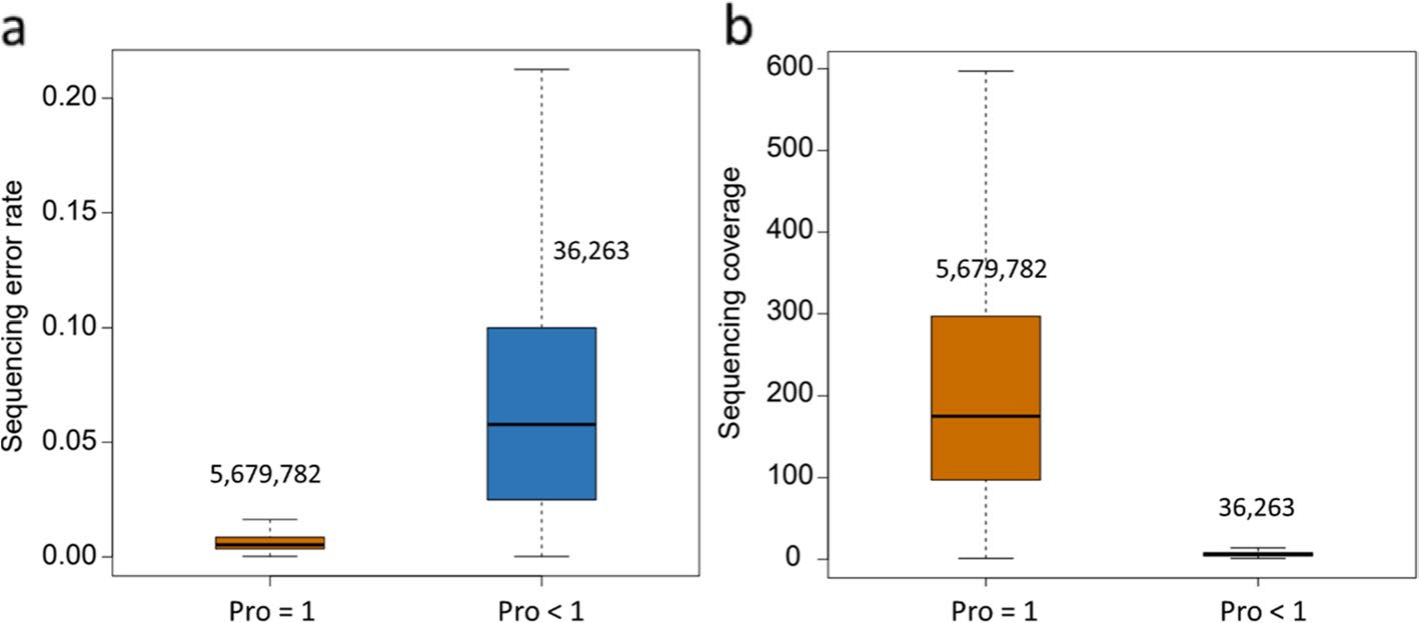
Comparison of sequencing error and coverage between high and low probability variants. **a** Sequencing error rate; **b** Sequencing coverage

The probability of each SNP for individual sample were calculated by incorporating the sequencing error rate (Fig. 1a). The probabilities of all SNPs from 5,061 sheep were divided into two groups: Pro = 1 and Pro < 1, and there were 5,679,782 and 36,263 variants in these two groups, respectively. The sequencing error rate of these two groups were significantly different, where the sequencing error rate of base in the high probability group was lower than that in low probability group (Fig. 3a). The high base sequencing error result in the low poisson probability of identified variant and make it unreliable. Conversely, the variant identified from the low sequencing error bases was more reliable (Fig. 3a). Further, the sequencing coverage (depth) of variants in high probability group were significantly higher than that in low group (Fig. 3b). Moreover, we further compared the identified variants with and without using poisson model. The variants number from with poisson model were less than that from without poisson model in majority samples, even the variants from 1,487 to 2,323 samples in with poisson model were all identified in without poisson model in Freebayes and GATK, respectively (Table S2). The unique variants from with poisson model were filtered in without poisson model due to the low mapping quality. Conversely, the unique variants from without poisson modes were identified by the higher mapping quality than these in with poisson model. Actually, the Alt-read number in uniquely identified variants of with poisson model was higher and more reliable than the unique variants from without poisson model (Table S3). Above result confirmed the necessary and rationality to consider the effect of sequencing errors in the identification of variants.

### The improvement of genetic variants identification

**Fig. 4:**
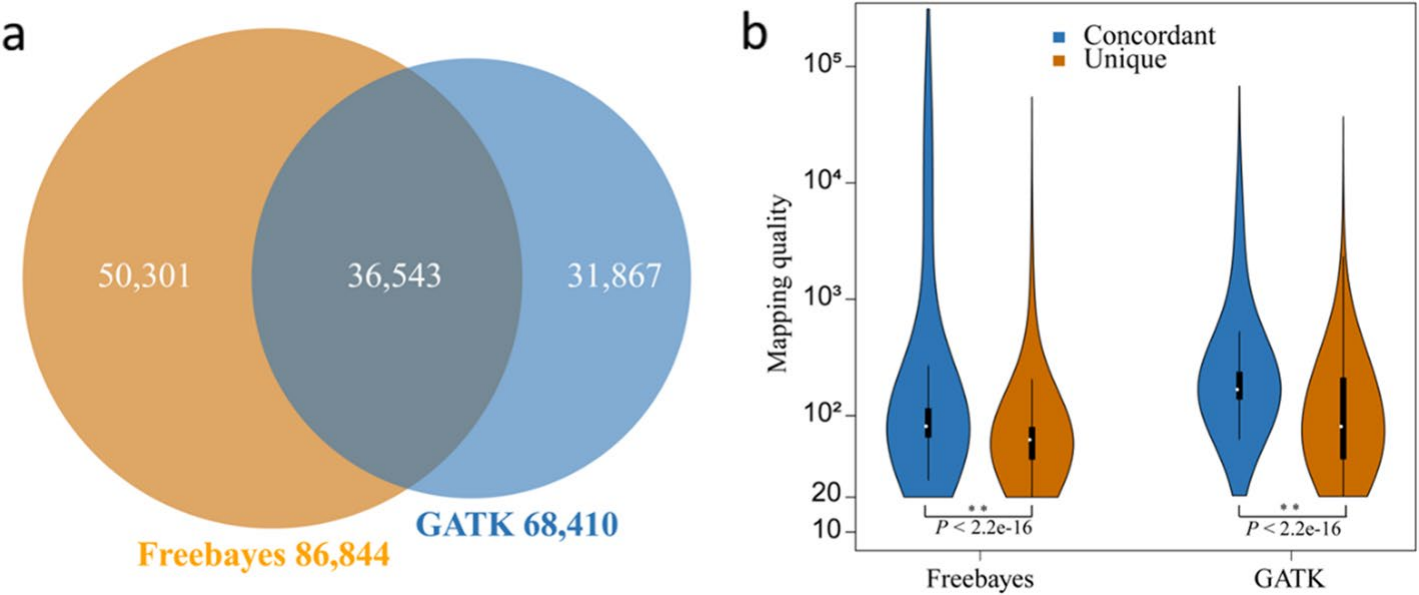
Concordance of variants identified using Freebayes and GATK. **a** Raw number of variants; **b** Comparison of concordant and unique variants in Freebayes and GATK

A total of 8,299,842 and 3,545,335 raw variants were identified in Freebayes and GATK, respectively. To validate the improvement of variants identification, the variants with mapping quality > 20 and sequence coverage > 5 were selected and compared with the FIV. After initial quality and depth control, there were 86,844 and 68,410 variants identified in Freebayes and GATK, respectively, and 36,543 of them were simultaneously identified in both callers that accounted for 42.08% and 53.42% in Freebayes and GATK (Fig. 4a). The mapping qualities of concordant variants (36,543) were significantly higher than unique variants identified in Freebayes (50,301) and GATK (31,867) (Fig. 4b). The confidence of the common variants identified from both callers were higher than the variants only identified in one caller, which provided the basis and evidence for constructing rHID dataset from both callers in FDR control procedure. The raw total variants identified in all 5,061 sheep included 48,439 SNPs and 31,373 Indels in Freebayes, and 41,773 SNPs and 28,992 Indels in GATK. The concordance of SNPs and Indels are 46.61% and 23.41% in Freebayes and 65.20% (SNPs) and 25.33% (Indels) in GATK (Table 1 and Fig. S2).

**Table 1:** Comparison of identified variants from Freebayes and GATK

Var type	Var source	Raw (%concordance)	FIV (%concordance)
SNP	Concordant	27,236	3,653
	GATK	41,773 (65.20%)	4,705 (77.64%)
	Freebayes	58,439 (46.61%)	4,651 (78.54%)
Indel	Concordant	7,345	1,935
	GATK	28,992 (25.33%)	3,703 (52.25%)
	Freebayes	31,373 (23.41%)	5,218 (37.08%)

The concordance of identified variants from Freebayes and GATK were compared between raw variants and FIV. There were 4,651 and 4,705 SNPs of FIV identified in Freebayes and GATK when incorporating multiple sample information, and the concordance of SNPs were 78.54% and 77.64% in Freebayes and GATK, respectively (Table 1). The percent of concordant SNPs and Indels from Freebayes and GATK in FIV were significantly improved 12%−32% compared with raw variants (Table 1). For each sample, the raw and final average SNP number were 1,118/1,296 and 1,079/1,268 in Freebayes/GATK, respectively (Table S4). The average concordance of SNP from 5,061 samples were increased from 94.13% to 96.33% in Freebayes and from 81.12% to 81.94% in GATK (Table S4). Moreover, the total identified number of SNPs and Indels in FIV variants were almost ten times less than raw variants, but the SNPs and Indels number of each sample were reduced by very little, which indicated the high confidence variants can be cross identified in multiple samples and low confidence SNPs uniquely identified in samples were effectively removed in the new strategy.

### Comparison of variants in FDR control process

**Fig. 5:**
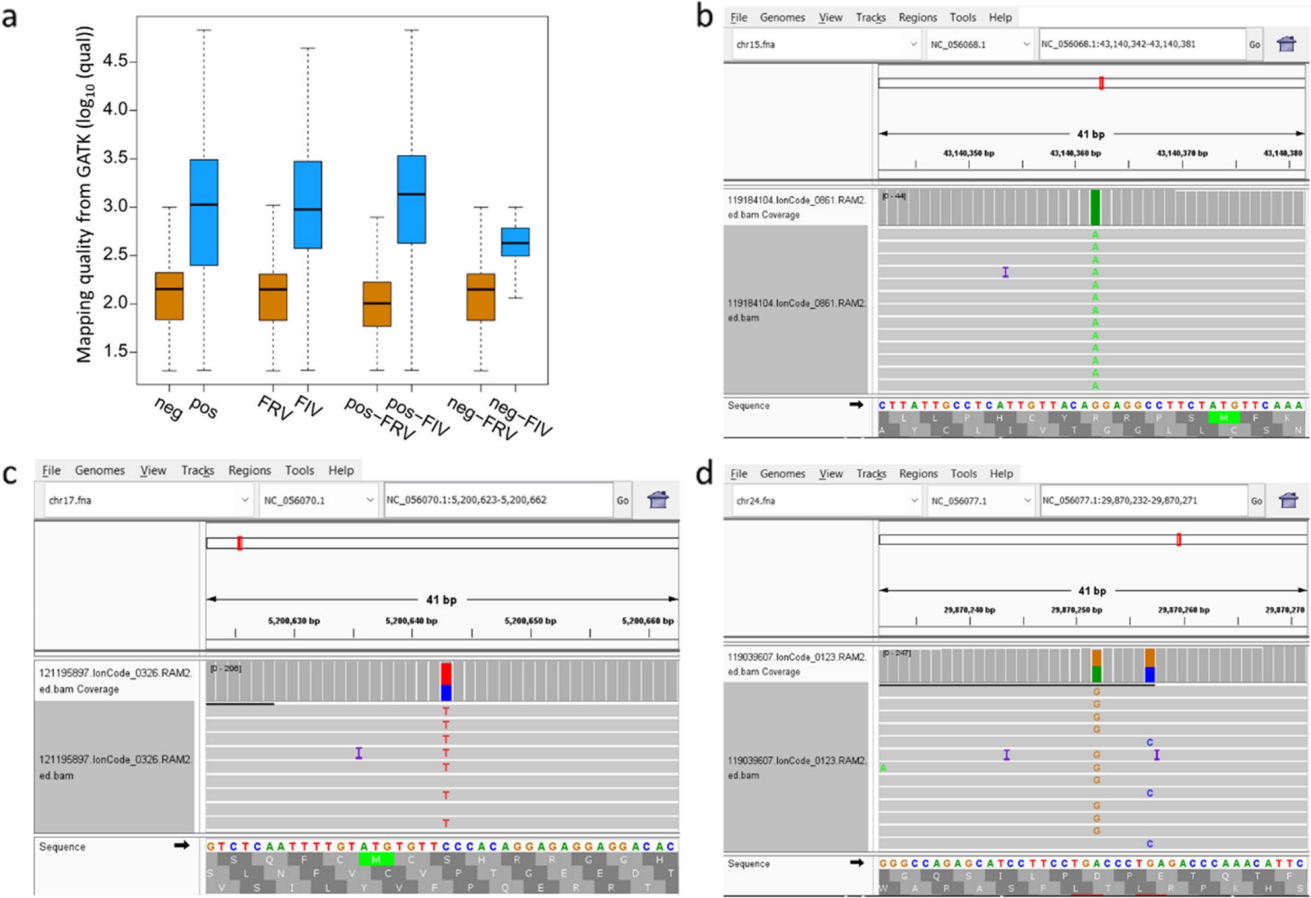
Comparison of mapping qualities from GATK and representative genes in IGV. **a** Mapping qualities in FDR control; **b** Confirmation of homozygous variant in *DENND5A*; **c** Heterozygous variant in *TMEM154*; **d** *GALNT17*

There were 128,931 variants for 5,061 sheep identified using Freebayes and GATK, and 9,979 and 118,952 variants were determined positive and negative according to the rHID database (Table S5). The location of positive or negative positions were intersected with variants of each sample and obtained FIV by using FDR control. The identified variants number in eight groups were: 118,952 (raw negative), 9,979 (raw positive), 10,399 (FIV), 118,532 (FRV), 8,194 (pos-FIV), 116,747 (neg-FRV), 1,785 (pos-FRV), 2,205 (neg-FIV) (Fig. 5a and Table S5). In the comparison of eight groups, the mapping qualities of positive dataset from rHID and three FIV datasets were significantly higher than negative and FRV datasets (Fig. 5a, Fig. S3 and Table S5). The FIV variants from raw positive group (pos-FIV) had the highest mapping quality that was significantly higher than pos-FRV group, which indicated that the FIV variants after FDR control were improved and more accurate. Furthermore, the rescued variants from neg-FIV group also had significantly higher mapping qualities than that in neg-FRV group (Fig. 5a and Fig. S3). In FIV dataset, although the total identified variants (10,399) from pos-FIV group (8,194) and neg-FIV group (2,205) were more than the raw positive dataset (9,979), the mapping quality between FIV and positive were not significantly different (Fig. 5a and Fig. S3), which further confirmed the FDR process effectively controlled the false positive and improved the genetic variants identification of multiple samples.

### Identification of rare frequency variants in 5,061 sheep

The total identified 10,301 variants were annotated by gtf annotation file from NCBI. There were 166, 9,362, and 773 variants in high, medium, and low groups, respectively (Table S6). A total of 166 variants from 102 genes with high frequency were identified in more than 90% of all samples (4,555). The variant (NC_056055.1: 7,483,382) at *PAPPA* gene of chromosome 2 had the highest frequency in all samples (5,053) (Table S6). Moreover, we identified 773 rare variants in individual samples, and 14 variants from 10 genes were with mapping quality > 1,000 and identified by both Freebayes and GATK callers. All 14 variants, including 11 heterozygous and 3 homozygous genotypes, were confirmed by IGV, and we presented the variants with the minimum coverage of heterozygous (69) (Fig. 5b) and homozygous (40) in IGV (Fig. 5c and d). The related genes with 14 variants have been previously reported in biological functions including nipples number, sub-vital white case, scrapie pathology, seasonal reproduction and litter size, coat color, congenital myotonia, and lentivirus susceptibility (Table 2).

**Table 2:** The variants of single sheep sample

Chr.	Pos.	Ref	Alt	Quality	Genotype (Coverage)	Genes	Traits	References
Chr 3	203,630,734	T	A	1,292.64	0/1 (87)	*ETV6*		
Chr 4	107,926,030	G	A	1,085.64	0/1 (110)	*CLCN1*	Congenital myotonia	[36]
Chr 7	53,213,797	C	T	1,097.64	0/1 (89)	*RAB27A*	Coat color	[37]
Chr 7	97,727,843	T	C	1,460.64	0/1 (144)			
Chr 8	25,802,309	C	T	1,545.06	1/1 (48)			
Chr 8	70,844,812	C	T	1,417.64	0/1 (97)	*GRM1*	Seasonal reproduction and litter size	[38]
Chr 10	66,396,204	G	A	2,214.64	0/1 (140)	*GPC5*	Number of nipples	[39]
Chr 15	43,140,362	G	A	1,086.06	1/1 (40)	*DENND5A*		
Chr 17	5,200,643	C	T	1,041.64	0/1 (69)	*TMEM154*	Lentivirus susceptibility	[40]
Chr 21	14,920,907	C	T	1,047.86	0/1 (298)	*TENM4*		
Chr 22	9,142,767	A	C	1,549.64	0/1 (98)	*PAPSS2*	Scrapie pathology	[41]
Chr 24	21,211,416	T	C	1,369.64	0/1 (75)			
Chr 24	29,870,252	A	G	1,064.64	0/1 (69)	*GALNT17*	Milk oligosaccharides synthesis	[42]
Chr X	92,508,011	T	A	2,366.06	1/1 (77)			

## Discussion

An accurate and comprehensive identification of genetic variants between one sample and the reference sequence is the major challenge in many studies [43–45]. Accurate identification of the variants from multiple samples in population scale can provide the foundation to stimulate the discovery of novel insights and a more accurate understanding of the biological mechanisms [25, 45, 46]. Possible reasons for unreliable variants identification are sequencing errors [20, 21], low-quality alignments [18, 19], or samples bias [17]. Our results confirmed that the base sequencing error generated by the sequencing instrument affects the accuracy of genetic variants identification (Fig. 2), which requires to consider the influence of sequencing errors in the identification of mutation sites. Calling as many potential true variants as possible and eliminating false positive variants are important ways to improve the accuracy of genetic variants identification. In this study, we conducted three measures to remove unreliable variants and obtain high confidence variants. First, sequencing errors were incorporated into the identification of SNP variants. Second, the mapping quality and consistent sample number from multiple samples were used to construct the positive dataset rHID for FDR control. Third, rescue the true negative variants by using the distributions of mapping qualities and consistent sample number from all 5,061 sheep data. The new method used the computational strategy to reduce the number of false positives, and simultaneously improve the identification of genetic variants (Fig. 5a; Table 1). This strategy didn’t incur any extra cost by using any additional samples or sequencing data information and was the best trade-off between accuracy and knowledge samples’ information for improving genetic variants identification in population scale.

The accuracy of identification of SNPs and Indels can be quantified by the number of true positives (TP), true negatives (TN), false positives (FP), and false negatives (FN) [33]. In this study, we not only assess the new method by using pos-FIV, neg-FIV, pos-FRV, and neg-FRV as the TP, TN, FP and FN, respectively, but also use rescue step to increase the true negative variants. The pos-FIV and neg-FIV were both significantly higher than pos-FRV and neg-FRV groups (Fig. 5a), and the total FIV variants (10,399) from pos-FIV group (8,194) and neg-POS group (2,205) were more than the raw positive dataset before FDR control (9,979), which illustrated the rescued variants are effective and indeed improved the genetic variants identification. Furthermore, despite the sharp decrease in the total identified SNP number from 27,236 to 3,653 from all 5,061 samples, the average SNP number of each sample only decreased 39 and 28 in Freebayes and GATK, respectively, which also confirmed that the high confidence variants cross identified in most samples were finally listed in FIV and most unique low confidence variants from individual were removed. Furthermore, the identified variants with at least 1×, 2×, 4× and 5× read coverages were assessed and compared in 5,061 sheep. For the raw variants from GATK and Freebayes, the variants number was decreased with the increasing of minimum coverage of read for the identified variants from 1× to 5×, but the FIV variants from the new strategy that optimized the variants identification by using multiple sample information was relatively stable (Table S7 and Table S8). The average and total identified variants were significantly less affected by the read coverage than these in raw identified variants (Table S8), and most of variants had a high concordant rate in all 1×, 2×, 4× and 5× data (Fig. S4). All above assessments and comparisons indicated that some low-confidence variants were effectively filtered by the new strategy, and the remaining variants were accurate.

The VQSR was used to recalibrate the combined VCF file after merging 5,061 samples by VariantRecalibrator and ApplyVQSR. Because there is no resource set for the non-human genome. The concordant variants from GATK and Freebayes were used to generate the resource set to conduct VariantRecalibrator in our study. The full procedure pipeline and related parameters can be found on github. A total of 3,828 variants of 73,614 in GATK were not “PASS” after VQSR, and only 375 variants that qualities were higher than 1,000 (Table S9). Besides, only 194 of 375 variants were identified in both GATK and Freebayes. The VQSR result confirmed that the rHID construction used to improve variant identification was reasonable. In order to identify more low frequency variants from multiple samples, the initial filter condition for variants should be moderately loose. If we initially filter the raw variants by VQSR, some variants will be losted in rHID construction. In our study, we list the VQSR value in one column of FIV and users can decide to keep the variant or not according to their actual situation.

The collected data were from the Flock54℠ program (https://www.flock54.com), which was created by Superior Farms in coordination with the University of Idaho and aimed to promote the usage of marker assisted selection in breeding [28]. The key component for genotyping germplasm is finding DNA sequence polymorphisms and assaying the markers across a full set of samples, and the excellent germplasm resources can be used as breeding materials [47]. Some investigations on decoding sheep traits were beginning to emerge from whole genome-wide sequencing and association studies, such as productivity [48], wool and skin [49–51], weight [52], preweaning growth [53], and disease resistance [54]. However, studies attempting to understand the impact of rare or less common variation on sheep breeding traits and diseases remain relatively limited. In this study, we identified the common and rare variants from 5,061 samples and found that low frequency variants of individual sheep involved several traits including nipples number (*GPC5*), scrapie pathology (*PAPSS2*), seasonal reproduction and litter size (*GRM1*), coat color (*RAB27A*), and lentivirus susceptibility (*TMEM154*). Although these genes had been reported to be associated with the traits, the rare variants were novel and identified with the benefit of the new strategy for calling variants from multiple samples in sheep. These rare variants in genes associated with these traits have the potential to contribute to breeding in sheep or other animals.

The genetic variant calling of SNP and Indel are problematic in population scale, as the exact variant types of the same position can be inconsistent among samples. The problem of the mixed variants calling was resolved by three steps in the new method. Firstly, the variants were discovered in each sample by GATK [11–13] and Freebayes [14], and the variants were split into separate SNP and Indel files. Then the Poisson probabilities of SNPs incorporating sequencing error were calculated and controlled by FDR to genotype the accurate SNPs using positive dataset from multiple samples information. For identification of genetic variants in population scale, the same variant may be simultaneously called as a SNP or Indel among different samples or different callers. we marked the variant types and reported all samples variants information without removing any variant types in the final list. The specific and detailed variant type in which sample requires user to further check and confirm based on their biological data and scientific problem.

We introduced a new computational method to identify genetic variants in targeted deep sequencing data from 5,061 samples in population scale, which improved variants identification by using the information of multiple samples. This strategy is not only for the targeted sequencing data but also efficient for WGS data. Here, we used 5 WGS sheep data from our lab to assess the applicability of the new strategy. The variants from longest chromosome (chr1) and computation time were presented and compared among GATK joint calling, Freebayes joint calling and new method (Table S10 and Fig. S5). Although the combined variants of 5 samples identified from new were less than GATK and Freebayes, the reduced number of variants in individuals is significantly less than that in total variants from combined VCF. Moreover, 92.88% of variants (1,726,518) from the new method were also identified in GATK or Freebayes, and the mapping quality of uniquely identified variants (635,119) from both GATK and Freebayes was significantly lower than the identified variants from all 3 lists (Fig. S5a and b). These results indicated that the low-quality variants were removed and high confidence variants were retained in the new strategy, which was consistent with the results from the sheep targeted sequencing data. Besides, the computational time of new method was between Freebayes and GATK. The most time used in GATK joint calling for variants was the HaplotypeCaller and GenotypeGVCFs due to the multiple samples. The time spent increases significantly as the sample size increases. Because new method separately called variants for each individual, so the spent time in HaplotypeCaller and GenotypeGVCFs is obvious less than that in GATK. The most time spent in new method is the poisson probability calculation from base sequencing quality and rHID construction. So far, the new method has only been evaluated in sheep. Theoretically, it is also suitable for other animals or plants population data, and the population-scale whole-genome resequencing data from other species needs to be investigated in future.

## Conclusions

With the drastically decreasing cost of high throughput sequencing, Pan-genomics is recently emerging and facilitates a more comprehensive characterization of genetic variation in population-scale. In this study, we developed a computational framework for joint calling genetic variants from 5,061 sheep by incorporating the sequencing error and optimizing mutual support information from multiple samples’ data. The percent of concordant SNPs and Indels from Freebayes and GATK after our new method were significantly improved 12%−32% compared with raw variants and advantageously found low frequency variants of individual sheep involved several traits including nipples number (*GPC5*), scrapie pathology (*PAPSS2*), seasonal reproduction and litter size (*GRM1*), coat color (*RAB27A*), and lentivirus susceptibility (*TMEM154*). The new method used the computational strategy to reduce the number of false positives, and simultaneously improve the identification of genetic variants. This strategy did not incur any extra cost by using any additional samples or sequencing data information and was the best trade-off between accuracy and knowledge samples' information for improving genetic variants identification in population scale.

### Supplementary Information

**Additional file 1: Fig. S1.** The distributions of mapping qualities and consistent sample number in GATK and Freebayes. **Fig. S2** The concordance of raw variants in SNP and Indel. **Fig. S3.** Comparison of mapping qualities at pre- and post-FDR in Freebayes. **Fig. S4.** Variants comparison in 1×, 2×, 4× and 5× coverage. **Fig. S5.** Variants venn of GATK joint, Freebayes joint and new method for WGS data

**Additional file 2: Table S1.** Mapping stats of 5,061samples

**Additional file 3: Table S2.** Poisson comparison of 5,061 samples

**Additional file 4: Table S3.** Poisson comparison of individual sample

**Additional file 5: Table S4.** Concordant variants in Freebayes and GATK

**Additional file 6: Table S5.** FDR control result

**Additional file 7: Table S6.** Final identification variants list of 5,061 samples

**Additional file 8: Table S7.** Comparison between raw and FIV in 1×, 2×, 4× and 5× of 5,061 samples

**Additional file 9: Table S8.** Average and total identified variants of 5,061 samples

**Additional file 10: Table S9.** VQSR results of GATK

**Additional file 11: Table S10.** WGS data result

## Abbreviations

FDR: False discovery rate
FIV: Final identified variants
FN: False negatives
FP: False positives
FRV: Final removed variants
FVar: Failed variants
GATK: Genome Analysis Toolkit
GFF: General feature format
G2P: Genome to phenome
IGV: Integrative Genomics Viewer
Indels: Insertions/deletions
MQ: Mapping quality
NGS: Next generation sequencing
rHID: Raw high-confidence identification database
RVar: Reliable variants
SN: Sample number
SNP: Single nucleotide polymorphism
TN: True negatives
TP: True positives
VCF: Variant call format
