# Supplementary material for: A computational framework for improving genetic variants identification from 5,061 sheep sequencing data
Source: J Anim Sci Biotechnol. 2023 Oct 2;14:127. doi: 10.1186/s40104-023-00923-3 (PMC10544426; doi:10.1186/s40104-023-00923-3)
Supplement: Supplementary file 1 — Additional file 1: Fig. S1. The distributions of mapping qualities and consistent sample number in GATK and Freebayes. Fig. S2 The concordance of raw variants in SNP and Indel. Fig. S3. Comparison of mapping qualities at pre- and post-FDR in Freebayes. Fig. S4. Variants comparison in 1×, 2×, 4× and 5× coverage. Fig. S5. Variants venn of GATK joint, Freebayes joint and new method for WGS data [file 40104_2023_923_MOESM1_ESM.docx]

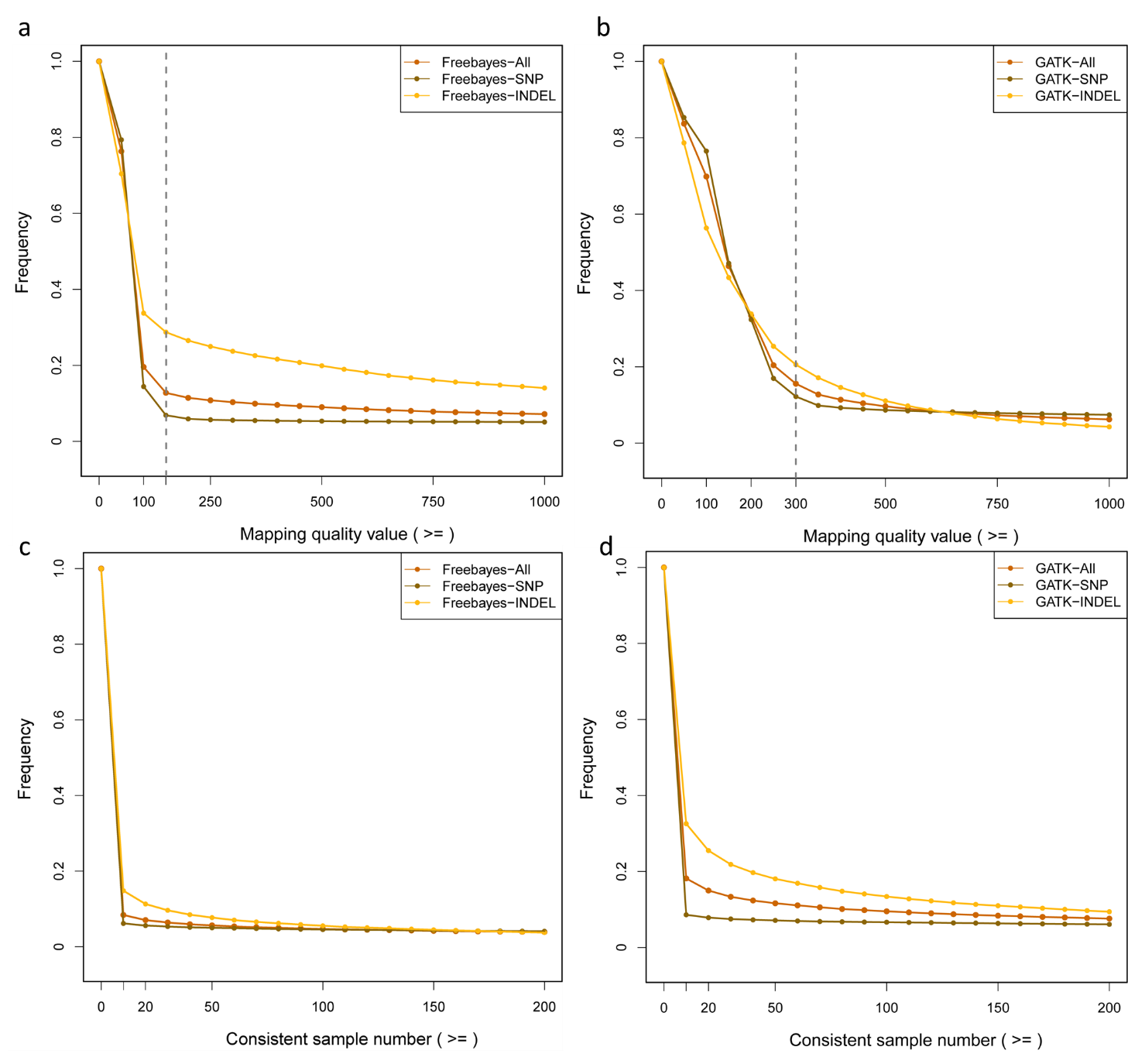


**Fig. S1** The distributions of mapping qualities and consistent sample number in GATK and Freebayes. **a** Mapping quality in Freebayes; **b** mapping quality in GATK; **c** consistent sample number in Freebayes; **d** consistent sample number in GATK


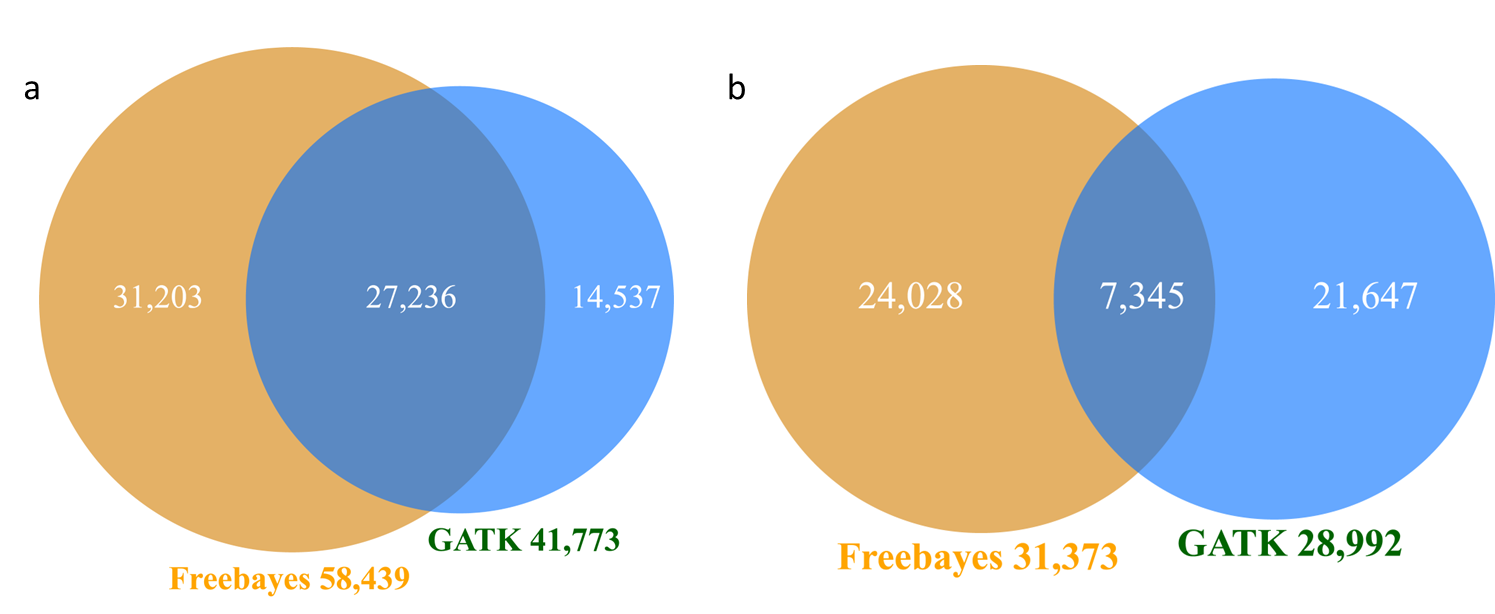


**Fig. S2** The concordance of raw variants in SNP and Indel from FreeBayes and GATK. **a** SNP; **b** Indel


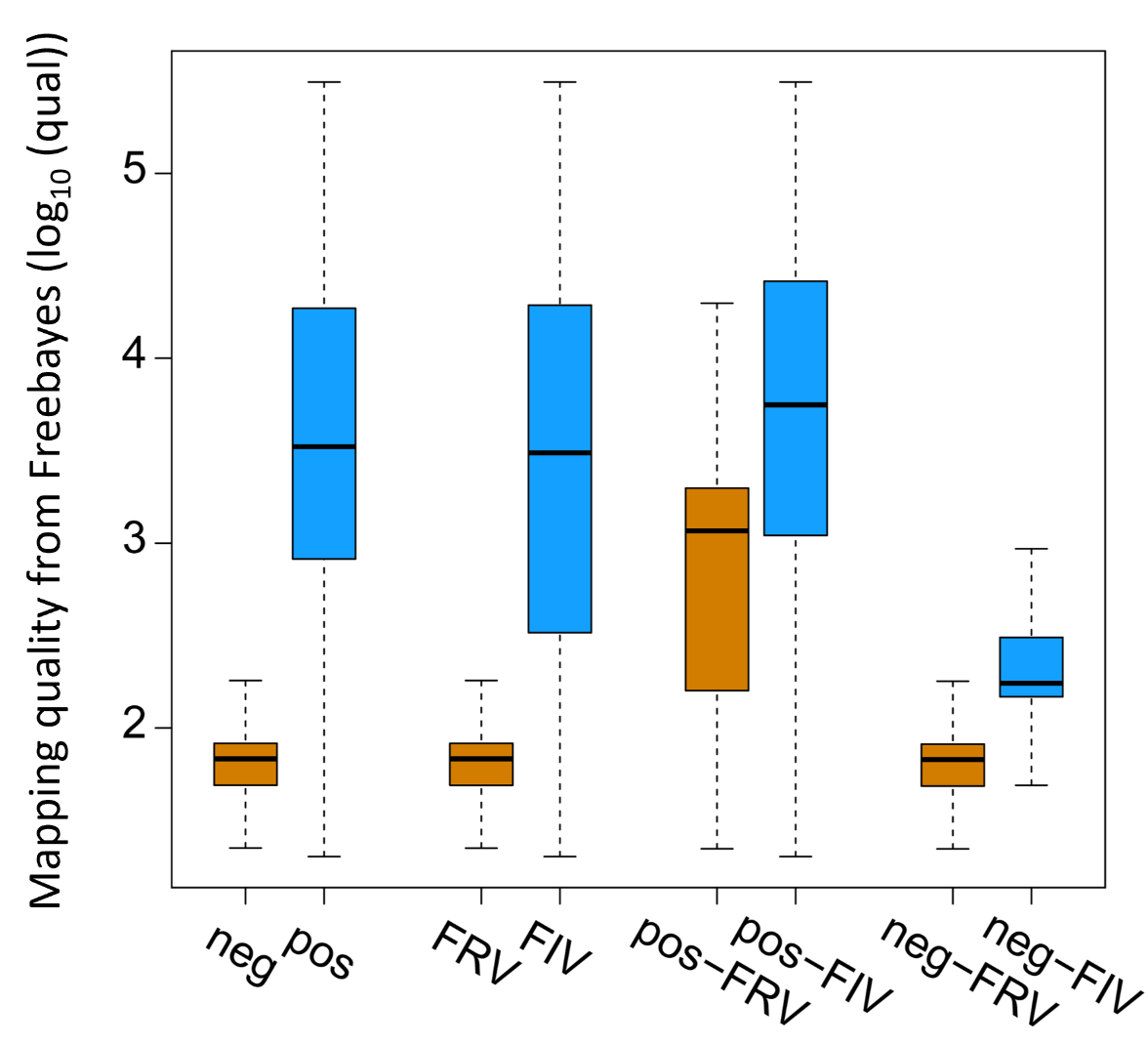


**Fig. S3** Comparison of mapping qualities at pre- and post-FDR in Freebayes

**
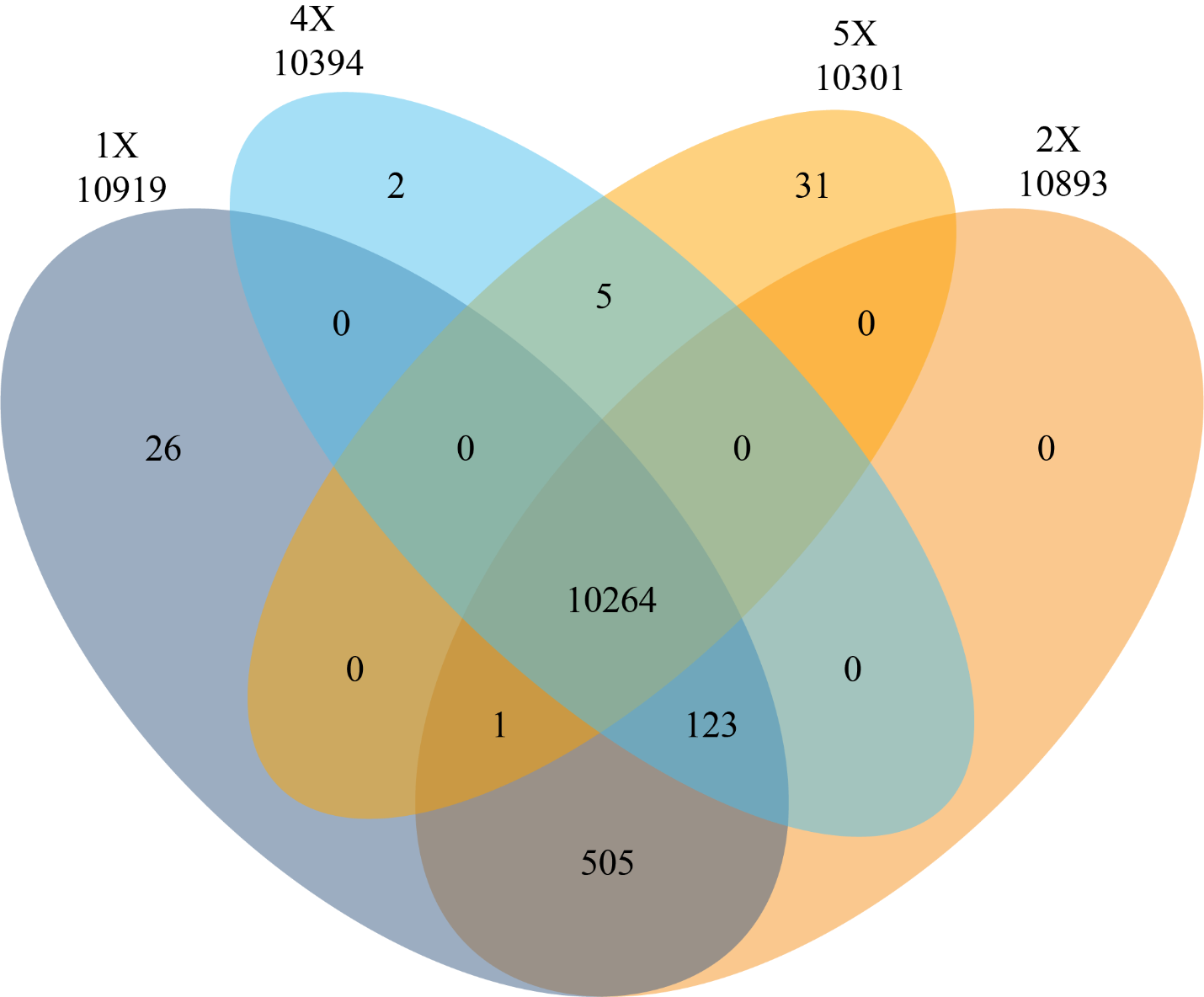
**

**Fig. S4** Comparison of variants with at least 1×, 2×, 4× and 5× read coverage in 5,061 sheep

**
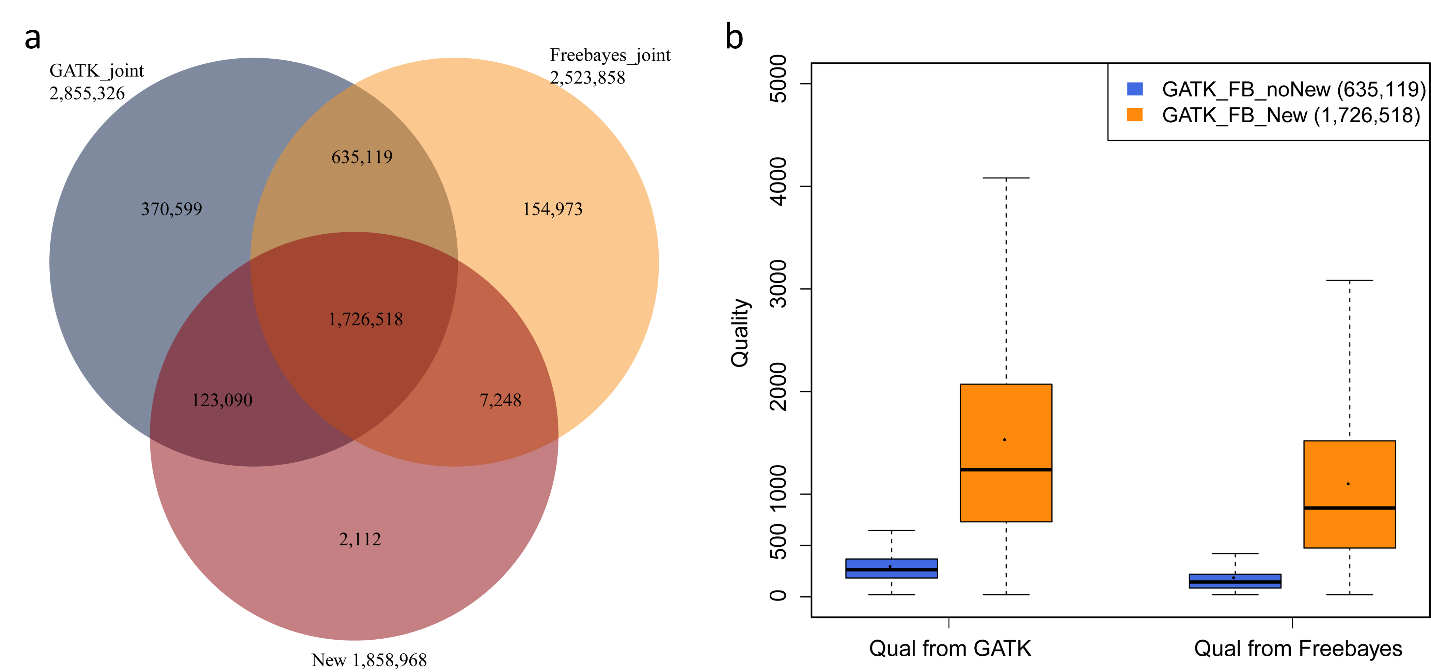
**

**Fig. S5** Comparison of new strategy with GATK and Freebayes joint variant identification in chr1 of WGS data. **a** Identified variants in three strategies; **b** comparison between the unique overlap from GATK and Freebayes and the overlap of all three strategies
